# Effectiveness of a Tailored Work-Related Support Intervention for Patients Diagnosed with Gastrointestinal Cancer: A Multicenter Randomized Controlled Trial

**DOI:** 10.1007/s10926-020-09920-z

**Published:** 2020-09-02

**Authors:** A. C. G. N. M. Zaman, K. M. A. J. Tytgat, J. H. G. Klinkenbijl, F. C. den Boer, M. A. Brink, J. C. Brinkhuis, D. J. Bruinvels, L. C. M. Dol, P. van Duijvendijk, P. H. J. Hemmer, B. Lamme, O. J. L. Loosveld, M. M. Mok, T. Rejda, H. Rutten, A. Schoorlemmer, D. J. Sonneveld, L. P. S. Stassen, R. P. Veenstra, A. van de Ven, E. R. Velzing, M. H. W. Frings-Dresen, A. G. E. M. de Boer

**Affiliations:** 1 Amsterdam UMC (Location AMC), Coronel Institute of Occupational Health, Amsterdam Public Health Research Institute, University of Amsterdam, Amsterdam, The Netherlands; 2 Amsterdam UMC (Location AMC), Department of Gastroenterology, University of Amsterdam, Amsterdam, The Netherlands; 3 Department of Surgery, Gelre Hospitals, Apeldoorn, The Netherlands; 4 University of Amsterdam, Amsterdam, The Netherlands; 5 Department of Surgery, Zaans Medical Center, Zaandam, The Netherlands; 6 Department of Gastroenterology, Meander Medical Center, Amersfoort, The Netherlands; 7 IKA- Ned, Hilversum, The Netherlands; 8 Department of Surgery, Northwest Hospital Group, Alkmaar, The Netherlands; 9 Department of Surgical Oncology, University Medical Center Groningen (UMCG), Groningen, The Netherlands; 10 Department of Surgery, Albert Schweitzer Hospital, Dordrecht, The Netherlands; 11 Department of Medical Oncology, Amphia Hospital, Breda, The Netherlands; 12 Department of Surgery, OLVG (Location East), Amsterdam, The Netherlands; 13 Tomas Rejda Counselling (Oncological Occupational Physician), Alphen aan den Rijn, The Netherlands; 14 Department of Surgery, Catharina Hospital Eindhoven, Eindhoven, The Netherlands; 15 Amsterdam UMC (Location AMC), Department of Surgery, University of Amsterdam, Amsterdam, The Netherlands; 16 Department of Surgery, Dijklander Hospital, Hoorn, The Netherlands; 17 Department of Surgery, Maastricht University Medical Center (MUMC), Maastricht, The Netherlands; 18 Department of Gastroenterology, Martini Hospital, Groningen, The Netherlands; 19 Department of General Surgery, Flevo Hospital, Almere, The Netherlands; 20 Vel.Onc@Work Counselling (Oncological Occupational Physician), Leidschendam, The Netherlands

**Keywords:** Return to work, Neoplasms, Randomized controlled trial, Vocational rehabilitation, Rehabilitation research

## Abstract

*Purpose* The aim of this research was to study the effectiveness on return to work (RTW) of an early tailored work-related support intervention in patients diagnosed with curative gastrointestinal cancer. *Methods* A multicenter randomized controlled trial was undertaken, in which patients were assigned randomly to the intervention or the control group (usual care). The intervention encompassed three psychosocial work-related support meetings, starting before treatment. Five self-reported questionnaires were sent over twelve months of follow-up. Primary outcome was days until RTW (fulltime or partial) and secondary outcomes included work status, quality of life, work ability, and work limitations. Descriptive analysis, Kaplan–Meier analysis, relative risk ratio and linear mixed models were applied. *Results* Participants (N = 88) had a mean age of 55 years; 67% were male and the most common cancer type was colon cancer (66%). Of the participants, 42 were randomized to the intervention group. The median time from sick leave until RTW was 233 days (range 187–279 days) for the control group, versus 190 days (range 139–240 days) for the intervention group (log-rank p = 0.37). The RTW rate at twelve months after baseline was 83.3% for the intervention group and 73.5% for the control group. Work limitations did statistically differ between the groups over time (p = 0.01), but quality of life and work ability did not. *Conclusion* Patients in the intervention group seem to take fewer days to RTW, albeit not to a statistically significant extent.

*Trial registration* Trial NL4920 (NTR5022) (Dutch Trial Register https://www.trialregister.nl)

## Introduction

The diagnosis of cancer has a major effect on a patient’s life. Daily functioning in normal activities, including work, is affected by (long-term) physical and mental health problems [1], e.g. fatigue [2–4], and cognitive problems [3, 5] due to the malignancy and its treatment. One of the challenges for patients treated with curative intent is their return to work (RTW) after the treatment. Work plays an important, positive role in people’s lives. It contributes to better quality of life [6], it gives the feeling of participating as a ‘normal’ individual i.e. structures everyday life [7–9]. Furthermore, work can provide a better self-image and esteem [10], it contributes to social inclusion [8] and offers a source of financial security [11]. This is becoming even more important as the number of patients diagnosed with cancer in the working population is rising, age at diagnosis is falling (due to screening programs) and with advanced treatments survival rates increasing.

Previous research has shown that patients experience work-related problems from the moment of diagnosis [12] and appreciate work-related information in the early stages of their cancer treatment [12–14]. Moreover, a longer absence from work is associated with a reduced probability of RTW [15, 16]. The health problems like fatigue and cognitive problems contribute to the RTW process and could even impede the work process. In the clinical setting, however, ‘work’ is not yet a standardized item discussed either in an early phase of diagnosis [17, 18] or later, once rehabilitation starts [19]. Yet it is relevant to prepare patients for the impact of cancer and its treatment on sick leave by giving them timely information about work-related issues, since this should enhance their self-empowerment to solve work-related problems. Therefore, the work-related information should be tailored to the patients’ needs and include for example; how to deal with openness about the diagnosis to colleagues and/or employer, information about the Act when patient is reporting for sickness absence, and disease- and treatment-specific factors in relation to work must be discussed [20]. There is an increasing focus on psychosocial and other forms of support, such as physical activity [5, 21] for patients diagnosed with cancer in the occupational and oncological context [5, 22]. Moreover, some intervention studies have focused on work- related support [23–25].

Currently, though, there is still no intervention that features early work-related support and is tailored to the needs of the patient. It was for this reason that we initiated the GIRONA study (Gastro-Intestinal cancer patients Receiving Occupational support Near and After diagnosis), providing tailored work-related support for patients diagnosed with GI cancer [20]. The intention of this intervention was to start informing patients about work-related issues in an early phase of their diagnosis and to support them if they experienced work-related problems. The intervention was tailored to the severity of their individual work-related problems and delivered by specific supporting healthcare professional.

Our aim was to study the effectiveness of the GIRONA intervention by performing a multicenter randomized controlled trial with a follow-up period of twelve months. The intervention was compared with the ‘usual’ clinical care of patients diagnosed with curative GI cancer, which involves no work-related support. The hypothesis was that offering tailored work-related support early in the clinical diagnostic phase would lead to enhancement of RTW and therefore result into fewer days of sick leave.

## Methods

The CONSORT statement [26, 27] was used to structure the trial methods and for reporting its results. In this paper we outline the tailored work-related support intervention itself; for background details, we refer readers to previous published articles on its design [20] and development [28].

### Trial Design

The study was designed as a parallel, multicenter randomized controlled trial (RCT) with a follow-up period of twelve months. The intervention included tailored work-related support for the intervention group, which was compared with ‘usual care’ received by the control group. The allocation ratio between the two groups was 1:1.

Supplementary to the previously published eligibility criteria [20], we adjusted one inclusion criterion. At the beginning of the study, this criterion was formulated as ‘patients were on sick leave at the time of diagnosis, i.e. the moment of study participation. As the study progressed, however, we noticed that patients were not always on sick leave yet at the moment of diagnosis, i.e. their first moment in the clinical setting. We therefore adjusted this inclusion criterion to assume that patients would be on sick-leave once their treatment started.

### Ethical Statements

The Medical Research Involving Human Subjects Act was applied for this RCT study, as confirmed by the Medical Ethics Review Committee of the Academic Medical Center (AMC), Amsterdam (registration number W14_248#14.17.0300). All participating hospitals approved local permission to start the study.

### Participants

The GIRONA study was conducted in 16 hospitals in the Netherlands. We started the study with fewer hospitals as described in the protocol [20], because several hospitals were pending for the local medical ethical approval to participate. The participants in the trial included healthcare professionals and patients. The oncologist and the oncological GI nurses recruited the patients. Oncological GI nurses and oncological occupational physicians (OOPs) were the professionals providing the work-related support. The OOP is a specialized occupational physician trained in supporting patients who are diagnosed with cancer and in dealing with work-related issues. The OOP works within the clinical setting or outpatient clinic. However, those OOPs are not yet officially incorporated in cancer care [29]. The patients included were persons diagnosed with a primary GI cancer (i.e. a malignancies in the digestive tract system, ranging from the esophagus to the colorectum) that was treatable with curative intent (all treatments were included). They were between 18–63 years of age and in paid work (including temporary and flexible contracts and self-employed) at the moment of diagnosis. It was originally intended that they would have to be on sick leave, either full or partial, at the time of diagnosis, but as mentioned this latter inclusion criterion was revised. Patients who had a severe mental disorder or other severe co-morbidities (observed by the nurses who included eligible patients, not from the self-reported patient questionnaire), were excluded. If there was a doubt about co-morbidities, this was discussed between research group and the nurse who included the patients.

### Enrollment and Informed Consent Procedure

The wards included were mainly surgical departments. One internal medicine department included patients, however, patients treated, for example, with neo-adjuvant therapy were discussed in the multidisciplinary meeting and therefore those patients could also be included by the oncological nurses. The nurses who enrolled the participants were also the nurses who provided the work-related support. Nurses were trained with a two-hour training before the start of the study, see for more details the design study [20].

Patients were asked to participate at the hospital where they were receiving their treatment. The oncological physician or nurse checked each patient’s eligibility by assessing the inclusion and exclusion criteria during their first visit to the hospital, then provided the patient with a brief explanation of the study. The patient was asked whether the researcher could contact them by telephone. If the patient agreed, they signed a specific informed consent form for telephone contact. They were also given a folder with an information leaflet, contact details and the informed consent form.

A member of the research team [AZ, SvH, FD, or LJ] then called the patient to provide more details about the study and to answer any outstanding questions. Written informed consent was obtained from all individuals who participated in the GIRONA study.

### The GIRONA Intervention

We developed this intervention [28] in close collaboration with a variety of healthcare professionals, including oncological physicians, oncological occupational physicians, oncological nurses and patients diagnosed with GI cancer. The tailored work-related support intervention encompassed three individual meetings of psychosocial work-related support. The first was to inform patients about work during and after treatment, to identify any work-related problems they might already have and to make a plan for their RTW or to stay in work. The first meeting was scheduled before the treatment began, the second a maximum of six months after the first and the third (if necessary) a maximum of nine months after the first.

Each meeting lasted approximately 30 min. The first and second meeting needed to be face-to-face as per protocol; the third could also be conducted by telephone. Topics discussed were the importance of work, contact with the work environment, transparency about their diagnosis with employer and/or colleagues, and the process of reporting sick under Dutch law.

The intervention itself was split into three types of work-related support (A, B and C); this was in order to respond to the patients’ individual needs, since work-related problems can differ in severity. Based on contributing factors to such problems as described in a decision diagram [28]. For example in support A; fatigue, pain and lack of support from family and friends, in support B; lack of support in work environment, neuropsychological problems and in support C; a combination of factors. These factors were assessed in the patients’ baseline questionnaire (T0). Based on the answers and the decision diagram, the researcher referred the patient to the tailored type of support, respectively A, B or C*.* Within each of these, the kind of healthcare professional assigned to provide supportive work-related care was tailored to the severity of the patient’s work-related problems and the healthcare professional takes the kind of work of the patient into account. In support type A this was an oncology nurse, in type B an OOP, and in type C a multidisciplinary team (including at least an oncology nurse, the treating physician, and an OOP). Details of the process evaluation are published separately [30].

### Usual Care

Standard or ‘usual’ psychosocial care is defined as care provided by the oncological nurse or oncological physician/surgeon, focusing on the treatment itself and related problems, e.g. pain or wound care. In general, work-related issues associated with the cancer diagnosis and treatment are not discussed.

### Measures

All the outcome measures were obtained from five self-reported patient questionnaires, completed on paper or in digital form at baseline (T0), three months (T1), six months (T2), nine months (T3), and twelve months (T4).

### Primary Outcome

The primary outcome was the number of days from sick leave until RTW (either fulltime or partial). Reflecting the Dutch social security system’s regulations in respect of sick leave, RTW was defined as the patient having returned to work for at least four successive weeks (regardless of their contract hours). The period between taking sick leave and RTW was measured using the dates (first day of leave and RTW) provided by the patient in the five questionnaires. When no first date of sick leave was reported, the fifteenth of the month of diagnosis was used; when no date of RTW was reported, the date of completing the questionnaire was used.

### Secondary Outcomes

Secondary outcome measures included employment status (RTW yes/no) at twelve months after baseline, as well as quality of life, work ability, and work limitations as assessed at each measurement point.

Quality of life was assessed using Short Form 12 (SF-12) [31, 32], covering twelve items (Dutch version). Questions inquired about the patient’s functional status, including their physical and social functioning, physical and emotional constraints, and general view of their own health. Their answers were converted into two summary norm-based scale scores, for physical and mental health respectively. In both cases, the higher the score, the better the respondent’s functioning, with a mean score of 50 (SD 10) in the general population [32]. SF-12 has been shown to be a valid and reliable instrument [31, 32]. Quality of life was additionally assessed with the EORTC QLC-C30, but we cannot report the results because of printing issues in the patient questionnaires.

Work ability was assessed using the first question from the Work Ability Index (WAI) [33] questionnaire (Dutch version), concerning current work ability compared with lifetime best ability. The answer format was a ten-point scale; the higher the score, the better the respondent’s work ability (0 = not currently able to work at all; 10 = work ability at its best). The WAI has been assessed as having good levels of reliability and validity [34].

Work limitation was measured using the Work Limitation Questionnaire (WLQ) [35], with a five-point scale evaluating the respondent’s functional limitations. The WLQ covers 25 items, aggregated into four subscales (time management, physical demands, mental-interpersonal demands, and output demands). The scale ranges from 0 to 100, with higher scores indicating more work limitations over the previous two weeks (0 = never limited; 100 = limited all the time). The English version of the WLQ has been shown to be valid and reliable when used for cancer survivors [36], has been translated into Dutch, and is valid and reproducible at group level among cancer survivors [37].

### Prognostic Factors

The following prognostic factors on RTW were taken into account: age, gender, marital status (married/cohabiting, single, divorced/widowed), educational attainment (low, intermediate, high), diagnosis, treatment type (not all patients had entered treatment at T0, therefore T1 was used), fatigue, depression, and cognitive functioning. Fatigue was measured using the Multidimensional Fatigue Inventory questionnaire (MFI), with 20 items divided into five subscales; subscale scores range from 4 to 20, with higher scores indicating greater fatigue [38]. Depression was measured using the scale developed by the Center for Epidemiologic Studies for depression (CES-D), with 20 items; scores range from 0 to 60, with higher scores indicating greater depressive symptoms measured for the past week [39]. Cognitive functioning was measured using the Cognitive Symptom Checklist-Work, Dutch Version (CSC-W DV), with 19 items; scores range from 0 to 100, with higher scores indicating more cognitive symptoms or limitations [40].

### Descriptive Factors

The descriptive factors included: main wage earner (yes/no), employment status (permanent, temporary, or self-employed), years in current position, and years in paid work. Another was workload perception, measured using the ‘physical workload’ subscale from the Perception and Judgment of Work questionnaire (VBBA), with seven items; scores range from 0 to 21, with higher scores indicating a higher physical workload [41]. Yet another was importance of work, measured using a Visual Analogue Scale (VAS); scores range from not important to most important. Also recorded was the use of other work-related co-interventions: a reintegration agency, reintegration coach, rehabilitation program, or support from other healthcare professionals.

### Sample Size

Data from a large occupational health service in the Netherlands was used [42] to estimate the RTW percentage in the control group at 63%. To estimate the RTW percentage in the intervention group, we referred to an earlier study in which cancer patients received a work-related support intervention including an educational leaflet and enhanced communications on the part of their attending and occupational physicians [43]. The RTW percentage in that study was 89% after twelve months; however, the median time between beginning treatment and enrollment was 42 days. Because our study started earlier, at the moment of diagnosis, we corrected this estimate with data from two previous studies conducted at our own hospital [44, 45]. The RTW percentage in these was approximately 8% in the first 42 days. Hence, the RTW for the intervention group in our study was calculated as 81% (= 89%—8%).

The required sample size was then calculated using the power and sample-size calculation program nQuery Advisor 7.0. A power of 80% and a p value of < 0.05 indicated that we should include a total of 216 patients, with a follow-up period of twelve months, to indicate a difference of 81% (intervention) versus 63% RTW (care as usual). Allowing for a 20% loss to follow-up and a 10% one-year mortality rate, 309 patients should therefore be included [20].

### Randomization

Once the researcher received their completed baseline questionnaire, the patient was randomized. Using a computerized web-based randomization program, ALEA [46], this randomization was conducted centrally at the AMC by the research team [AZ, SvH, FD, LJ, and AdB] for all participating hospitals. The biased-coin principle was used, with a threshold of two. As patients could differ between the participating hospitals in terms of diagnosis and demographic factors (e.g. age), and because these factors are important prognostic factors for RTW [47], randomization was stratified for gender, age (age groups: 18–54 and 55–63 years), and hospital, so as to prevent bias due to unequal randomization. Patients were numbered with three-digit sequential Patient Identification Numbers (PINs). Minimization was applied to equalize group sizes. These PINs were used on the patient questionnaires, which were collected by the research team [AZ, SvH, FD, LJ or AdB].

### Blinding

Patients, healthcare professionals, and researchers were not blinded for the group assignment. One of the researchers [AZ, SvH, FD, or LJ] contacted the healthcare professional and the patient after randomization, so that the first meeting could be scheduled (when patient was randomized to the intervention group). The unique PIN was used for blind analysis of the data by the researcher.

### Statistical Analysis

Data derived from the patient questionnaires was verified by means of a 20% double check. The primary outcome, RTW, was 100% double checked [AZ, AdB]. All patients were included in the analysis in accordance with the intention-to-treat (ITT) principle. However, patients who died during the study or follow-up period were excluded from the survival analysis. All analyses were performed using the statistical package IBM SPSS Statistics 25.

Possible differences between the intervention and control groups at baseline were verified using Student’s t-test for continuous variables and the χ^2^ test for categorical data. A p-value of ≤ 0.05 was considered as statistically significant. The primary outcome, days until RTW (either fulltime or partial), was analyzed using the Kaplan–Meier survival method and differences between the intervention and control groups were analyzed using the log-rank test. When the RTW event did not happen, i.e. the patient was no longer ‘at risk’ of RTW (no further data was available), then these patients were censored. A sensitivity analysis was performed excluding those who stopped participating in the study or were not reported as being on sick leave at any measurement point during the entire study period. After that, a Cox regression analysis was used to represent the estimated hazard ratio (HR) with 95% confidence interval for the time until RTW. The dependent variable was RTW at twelve months after baseline and the independent variable was group of randomization.

If there was a significant difference in the prognostic factors between the groups at baseline, then the primary outcome variable was adjusted for these factors in a multivariate Cox- regression analysis.

The relative risk (RR) with 95% confidence interval was presented for employment status at twelve months after baseline, comparing the intervention group with the control group.

Linear mixed models (LMMs) were applied to the longitudinal data to examine differences over time between the intervention and the control group in respect of the secondary outcomes (quality of life, work ability, and work limitations). In the LMM model, the baseline and three, six, nine, and twelve-month scores were included as dependent variables for quality of life and work ability. In the case of work limitations, scores at baseline were not available because patients had to be working then, so only three, six, nine, and twelve-month work-limitation scores were included in the LMM model. We included the fixed effects randomization group and time, and the interaction effect randomization group*time. The subjects were included as random effects. The interaction term (randomization group*time) indicated a difference in time on the outcome (quality of life, work ability, and work limitations) between the study groups. The effects of the study groups could be interpreted as a difference between the groups (control versus intervention) over the follow-up period. A p-value of ≤ 0.05 was considered as statistically significant. If there was a statistically significant interaction (randomization group* time) effect, then a T-test was applied per time measurement.

## Results

### Recruitment and Participant Flow

**Fig. 1:**
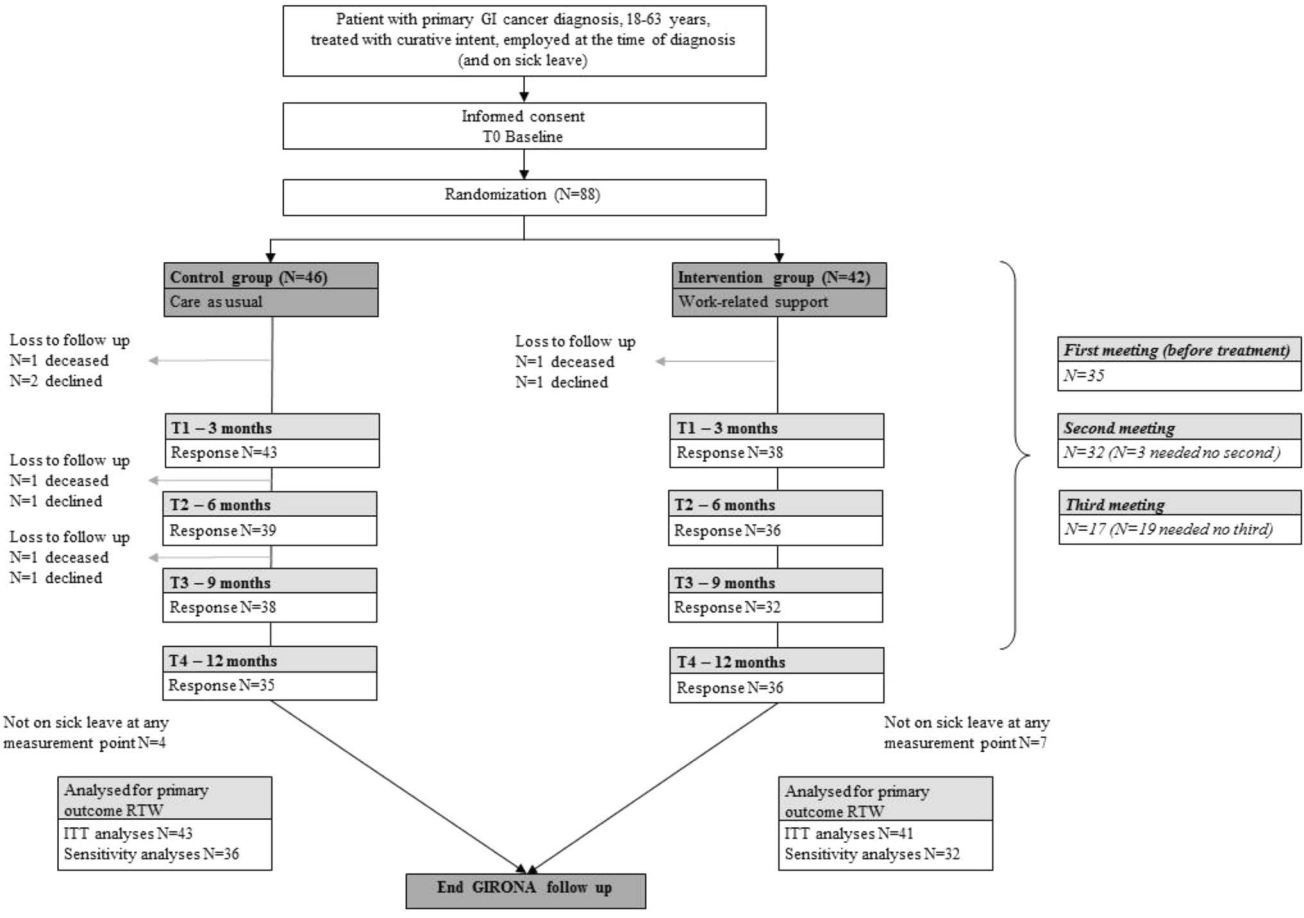
Participant flow diagram

Patients were enrolled in the GIRONA study between May 2015 and May 2017, the original period of recruitment May 2015–July 2016 was extend to May 2017 due to the slow inclusion rate. The follow-up period lasted twelve months. Figure 1 presents the participation flow. In total, 88 patients were included; of these, 46 were randomized to the control group and 42 to the intervention group. Accordingly, the baseline questionnaire was filled out by 88 participants (100%). The response rates for the remaining questionnaires were: T1 at three months, N = 81 (92%); T2 at 6 months, N = 75 (85%); T3 at nine months, N = 70 (80%); and T4 at twelve months, N = 71 (81%). At baseline, 64 patients were already on sick leave. Nine more reported having taken sick leave at T1 or T2, and eleven were not on sick leave at any measurement point during the study period.

Reasons for not returning one of more questionnaires included death (N = 4; three in the control group and one in the intervention group), withdrawal from the study (N = 5) or unknown despite reminders (N = 21).

### Patients’ Characteristics at Baseline

Table 1 summarizes the baseline participant characteristics for the intervention and control groups. Of the 88 patients, 67% were male (N = 58) with an average age of 55 years (SD 7.2 years). The most common type of diagnosis was colon cancer (N = 58; 66%), followed by rectal cancer (N = 18; 21%). There were no statistically significant differences at baseline between the intervention and control groups in any sociodemographic, clinical, work-related or health-related characteristic.

**Table 1:** Patients’ baseline characteristics

Patient characteristics	Intervention group N = 42^*^		Control group N = 46^*^		p value^**^
*Sociodemographic*					
Age (years)	54	± 7.7	56	± 6.6	0.26
Gender (% male)	64%		67%		0.76
Marital status					
Married/cohabiting	33		39		0.27
Single	8		4		
Divorced/widowed	1		3		
Main wage earner					
Yes	19		21		0.90
No, my partner is	3		4		
Equal with partner	18		17		
Gross monthly income					
≤ €1000	1		2		0.89
€1001–€2000	9		11		
€2001–€3000	15		20		
€3001–€4000	6		4		
≥ €4001	1		1		
Educational attainment					
Low	9		15		0.46
Intermediate	17		15		
High	16		15		
*Clinical characteristics*					
Cancer diagnosis					
Stomach	–		1		0.61
Liver	1		1		
Gallbladder	–		1		
Small intestine	1		–		
Colon	30		28		
Rectal	7		11		
Pancreatic	2		4		
Anal	1		–		
Treatment (T1 3 months)***					
None	0		1		0.32
Surgery (S)	18		21		
Chemotherapy (CT)	2		0		
Radiotherapy (RT)	1		0		
Combination (S-CT-RT)	21		16		
*Work-related characteristics*					
Occupational sector					
Healthcare/education	11		5		0.08
Administrative	–		6		
Sales	3		1		
Industry/transport/logistics	8		12		
Business services	13		14		
Other	7		8		
Years in current function	16.4	± 11.5	17.35	± 11	0.70
Years in paid work	31.3	± 11	32.8	± 9.9	0.50
Employment status					
Permanent	30		41		0.19
Temporary	3		1		
Self-employed	6		3		
Hours under current contract (per week)	37	10.8	35	9.3	0.49
Physical workload****					
VBBA *Score 0–28*	4.3	± 5.1	4.9	± 4.6	0.56
Importance of work****					
* Score 0–100*	51.6	± 29.5	47.7	± 29.2	0.53
Reconsider importance of work					
No	30	71%	27	60%	0.26
Support from family and friends					
No, no need of	16		17		0.56
No, but need it	1		–		
Yes, I have this support	25		29		
Support from work environment					
No, no need of	4		10		0.15
No, but need it	2		5		
Yes, I have this support	35		31		
*Health-related characteristics*					
Fatigue (general)****					
MFI *Score 0–20*	12	± 5.1	11.9	± 5.1	0.91
Depression****					
CESD *Score 0–60*	12.1	± 9.9	11.4	± 9.1	0.74
Cognitive functioning****					
CSCW-DW *Score 0–100*	21	± 15.5	27.1	± 18.8	0.1

^*^ Due to missing values or rounding differences, numbers may approach the total N and 100%

^**^Continuous variables mean ± standard deviation l Nominal and ordinal variables (N) with percentages. Student’s t test for continuous variables and χ^2^ test for ordinal and nominal variables

^***^Treatments are presented at T1 (3 month) because patients were included at the moment of diagnosis and therefore not available at T0

^****^The higher the score, the higher the level of physical workload, importance of work, fatigue, feelings of depression, and cognitive functioning problems

Treatments are presented at T1 3 month because patients were included at the moment of diagnosis and therefore not available at T0. At T1 80 patients filled out the question about the treatment they received so far; one did not had any treatment yet, 39 patients underwent surgery, two patients received chemotherapy, one patient had radiotherapy as treatment and 37 patients filled out that they received a combination of operation- chemo and radiotherapy.

### The Tailored Work-Related Support Intervention

Of the 42 patients who were randomized to the intervention group, two declined to take part in the intervention meetings before the first was planned, one died before the first could be planned, and one declined after a meeting was planned. Of the remaining 38 patients in the intervention group, 20 (53%) were referred to support type A (oncological nurse) and 18 (47%) to type B (OOP). None was referred to type C (multidisciplinary team). Further details of the intervention procedure outcomes are described in the process evaluation article (submitted for publication). No adverse events were reported, either by patients or by healthcare professionals.

### Primary outcome: time until RTW

#### Intention-to-Treat Analysis (ITT)

**Fig. 2:**
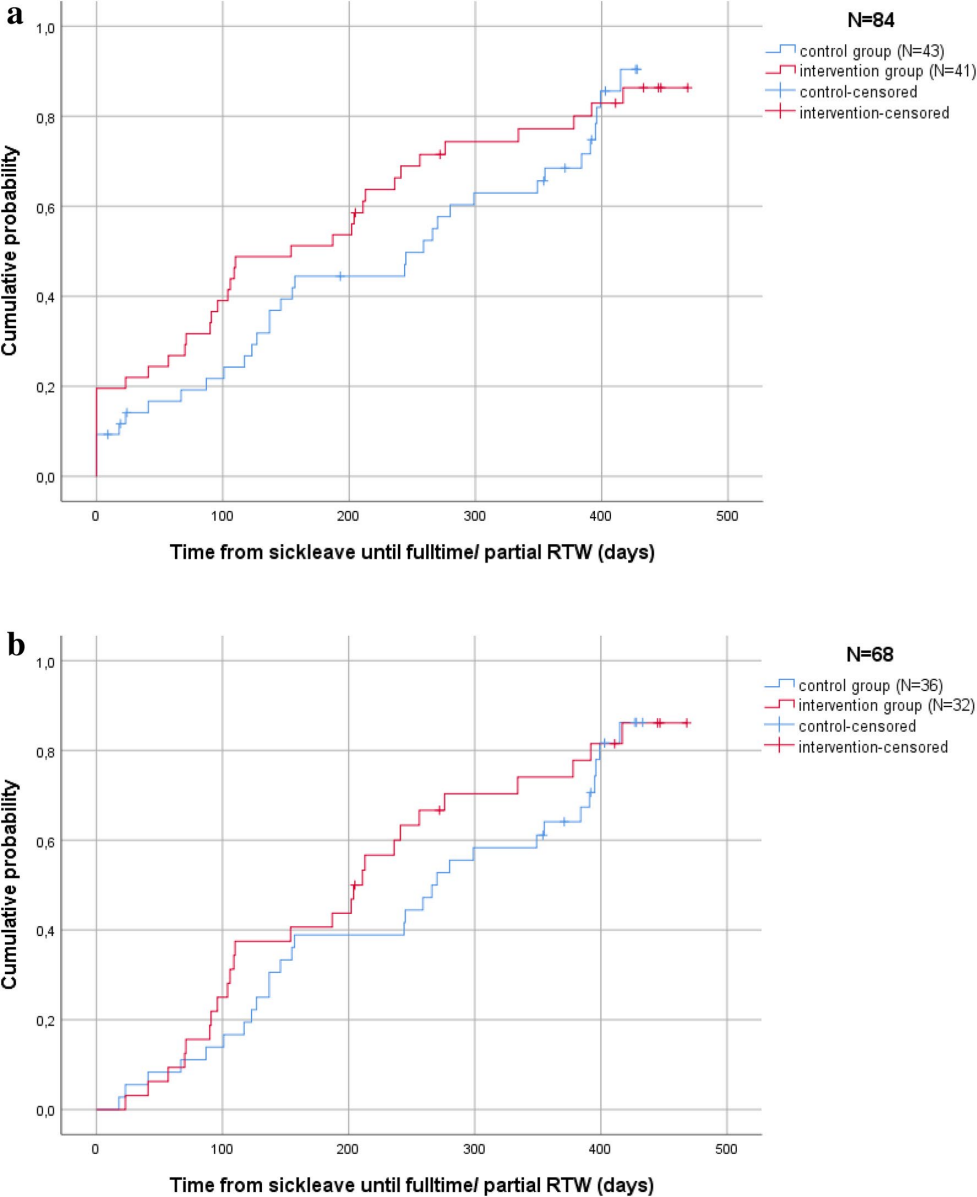
**a** Kaplan Meier (KM) survival ITT analysis for time until return to work (fulltime or partial).**b** Kaplan Meier (KM) survival Sensitivity analysis for time until return to work (fulltime or partial)

The median time from sick leave until fulltime or partial RTW was 233 days (187–279 days, 95% CI) for the control group and 190 days (139–240 days, 95% CI) for the intervention group. There was no statistically significant difference between these groups concerning time from sick leave until fulltime or partial RTW (log-rank p value = 0.37). Figure 2a shows the Kaplan–Meier survival analysis; the vertical line represents the 11 patients who were not on sick-leave at any time during the study, reflecting their period until RTW as 0 days (since the event ‘RTW from sick leave’ did not happen). The hazard ratio (HR) for RTW (either fulltime or partial) at twelve months after baseline was 1.2 (95% CI 0.77–2.0) for the intervention group versus the control group.

### Sensitivity Analysis

Figure 2b shows the subgroup Kaplan–Meier survival analysis, excluding patients who did were not on sick leave at any point between baseline (T0) and twelve months (T4) (N = 11) and those who stopped participating (N = 5). Patients who were randomized to the intervention group but declined to take part in the intervention received control-group questionnaires and were analyzed as control-group participants (N = 2). Those who died (N = 4) during the study were again excluded. The median time from sick leave until fulltime or partial RTW was 260 days (214–305 days, 95% CI) for the control group and 228 days (177–278 days, 95% CI) for the intervention group. This was not statistically significant (log-rank p value = 0.39).

### Secondary Outcomes

#### Relative Risk (RR): Returning to Work

The RTW (fulltime or partial) rate at twelve months follow-up, was calculated using the available data from 70 patients (intervention group N = 36, control group N = 34), was 83.3% for the intervention group and 73.5% for the control group (p = 0.32). The RR of returning to work at twelve months follow-up after baseline was 1.13 (95% CI 0.88–1.45) for the intervention group versus the control group.

#### Effects Over Time for Quality of Life, Work Ability, and Work Limitations

Table 2 presents the outcomes over time of quality of life, work ability, and work limitations. All of these secondary outcomes improved to a statistically significant extent over time. However, the main effects per group (intervention versus control) were not statistically significant for either component—physical score (PCS) and mental score (MCS)—of the quality of life outcomes, or for work ability and work limitations (respectively, PCS p = 0.09/MCS p = 0.45, work ability p = 0.14, and work limitations p = 0.33). The interaction effects of randomization group*time were not statistically significant (randomization group*time; PCS p = 0.59/MCS p = 0.13 and work ability p = 0.15). For both groups, the PCS had its lowest score at three months from baseline and had increased again at nine months from baseline.

**Table 2:** Secondary outcomes over time; quality of life, work ability and work limitations from baseline up to 12 months follow up, specified by intervention and control groups

	T0-baseline mean (SD)		T1–3 months mean (SD)		p value	T2–6 months mean (SD)		P value	T3–9 months mean (SD)		P value	T4–12 months mean (SD)		p value	p value LMM analyses		
	Intervention	Control	Intervention	Control		Intervention	Control		Intervention	Control		Intervention	Control		Group	Time	Randomization-group* time
Quality life																	
* PCS*	46.6 (10.0)	46.7 (9.9)	44.6 (11.2)	40.2 (9.9)		44.8 (10.7)	40.8 (12.0)		46.9 (8.3)	43.6 (10.9)		47.7 (9.5)	44.8 (11.1)		0.09	**0.00**	0.59
* MCS*	44 (9.9)	45.3 (9.8)	46.8 (9.8)	45.3 (9.2)		46.2 (10.7)	49.4 (8.3)		47.6 (8.8)	46.8 (10.4)		47.7 (9.7)	50.8 (7.5)		0.45	**0.00**	0.13
Work ability	5.1 (3.0)	5.3 (3.2)	5.7 (2.8)	4.1 (2.9)		5.4 (2.8)	4.5 (3.2)		6.5 (2.2)	5.5 (2.7)		6.5 (2.5)	6.1(2.1)		0.14	**0.00**	0.15
Work limitation^**^	–	–	13.3 (10.9)	23.5 (17.8)	**0.05**	15.8 (19.3)	19.2 (16.0)	0.55	17.7 (15.3)	16.7 (15.5)	0.83	17.2 (15.8)	16.7 (14.1)	0.90	0.33	**0.01**	**0.01**
* Time*	–	–	24.8 (26.3)	31.1 (24.8)		17.8 (23.3)	18.2 (21.6)		22.4 (24.5)	15.6 (20.6)		21.4 (24.3)	21.7 (23.6)		0.75	**0.00**	0.11
* Physical*	–	–	4.3 (8.3)	8.8 (14.0)		9.9 (12.8)	22.1 (32.2)		25.2 (34.5)	19.1 (25.9)		14.3 (27.1)	13.4 (16.9)		0.51	**0.05**	0.14
* Mental-interpersonal*	–	–	12.1 (11.3)	23.5 (17.1)	**0.03**	14.6 (21.8)	18.0 (16.4)	0.59	14.5 (17.4)	14.8 (14.6)	0.96	14.9 (17.2)	14.5 (12.1)	0.93	0.34	**0.00**	**0.02**
*Output*	-	-	11.9 (13.4)	25.1 (22.0)	**0.04**	19.0 (22.7)	19.0 (20.0)	0.99	14.4 (17.0)	19.2 (19.4)	0.38	18.6 (19.7)	18.1 (15.4)	0.92	0.42	**0.01**	**0.01**

Factors with a significant result are highlighted in bold

^*^Due to missing values numbers approach the total N

^**^Work limitations questionnaire was not applicable at baseline. The questions were filled out when participant was returned to work, therefore the numbers varied over time: T1 N = 35 (I = 19/C = 16) / T2 N = 39 (I = 20/ C = 19) / T3 N = 45 (I = 24/C = 21) / T4 N = 53 (I = 28/C = 25)

For the work limitations outcomes, the intervention group scored fewer limitations at both three and six months after baseline than the control group. The main effect over time was statistically significant (p = 0.01). On two (mental-interpersonal demands and output demands) of the four subscales of the work-limitations questionnaire, there was a statistically significant effect on the main interaction effect of randomization group*time (respectively, p = 0.02 and p = 0.01). This effect was statistically significant at measurement T1 after three months (WLQ general, p = 0.05; mental-interpersonal demands p = 0.03 and output demands p = 0.04).

## Discussion

The hypothesis of this study was that offering tailored work-related support early in the clinical diagnostic phase would lead to enhancement of RTW and therefore result in fewer days of sick leave. There was no statistically significant decrease in RTW, although numerically the intervention-group patients returned to work after a mean of 190 days of sick leave which a difference of 43 days compared with the control-group average.

The RTW rates at twelve months after baseline were relatively high for both groups. But again, neither this result nor the other secondary outcomes quality of life and work ability (outcomes between the groups over time) were statistically significant. Work limitations did show a significant result, in the sense that the intervention group had fewer work limitations at three months after baseline.

### Methodological Consideration

The major methodological concern, and the explanation for the lack of statistical significant differences between the groups, is the power of the study. Unfortunately, we were unable to include the number of patients prescribed under the predetermined sample-size calculation (309intotal). This was possibly due to other ongoing (interfering) studies, or to the timing of the study’s introduction to prospective participants (in the diagnostic phase). Based on previous studies, the RTW rate with ‘care as usual’ was 63%, compared with 81% for the group who received a work-related support intervention. We did reach those rates in this study, too (RTW at twelve months after baseline: control 73.5% versus 82.3% for the intervention group), but without statistical significance due to its insufficient power.

### Interpretation of the Findings

Despite the lack of statistically significant results, this study nevertheless produced some potentially promising outcomes. First, the number of days between taking sick leave and RTW was lower for those who received the work-related support intervention. These results are encouraging, as they were found at three to six months after baseline (see Fig. 2 and Table 2)—i.e., during the period in which the intervention was being performed. From the results of the process evaluation (submitted for publication), we know that the first meeting before treatment was difficult to conduct in practice, but these meetings were held as soon as possible and the second meetings were mostly performed within six months of the first.

Another promising finding of our study is that the RTW rates at twelve months after baseline are relatively high for both groups: 83.3% for the intervention group, compared with 73.5% for the control group.

The results of the systematic review conducted by Mehnert [48] underline these high RTW rates. That review reported a mean of 63% (range 50–81%) of participants managing to RTW or to stay in work during treatment at twelve months following the diagnosis of cancer. Our intervention was developed to support and inform patients about work, RTW, and related problems at an early stage. So although we expected the RTW rate of the control group to be lower than that of the intervention group, because they did not receive work-related support, its result is still a positive finding of this study. Despite the lack of work-related support, their RTW percentage showed to be relatively high by comparison with RTW percentage published in the review of Mehnert [48]. One possible explanation for this is raised awareness of the importance of work even among the control group in our study, because their mere inclusion in the study drew their attention to possible RTW problems—firstly because ‘work’ attracted more overall social interest during the period of the study. Secondly because all participants were informed about its aim before they signed their informed consent to participate, which could have resulted in ‘information-biased’ results. Moreover, the questionnaires sent to both groups, control and intervention, included work-related items. Patients were therefore constantly reminded about the importance of work. As a consequence, it could be that control-group patients themselves took action to seek the support they needed. As outlined earlier (Table 3), for example, control-group patients had paid twice as many visits to an occupational physician than participants in the intervention group at three months after baseline. So even though this may also explain the lack of statistically significant differences between the groups, we may assume that informing patients at an early stage triggered them to think about RTW. This further stresses the importance of including work-related information in day-to-day clinical practice.

**Table 3:** Work-related support other than the GIRONA tailored intervention, specified by intervention and control groups

	T1–3 months		T2–6 months		T3–9 months		T4–12 months	
	Intervention	Control	Intervention	Control	Intervention	Control	Intervention	Control
Work-related support outside the hospital^*^								
Reintegration agency	1	–	–	–	–	–	–	–
Reintegration coach	1	2	1	1	–	1	–	–
Rehabilitation program	3	1	1	–	4	4	3	1
Other^***^	6	2	1	6	3	5	4	4
Other healthcare professionals ^**^								
Occupational physician	12	24	13	17	10	18	10	13
Social worker	2	2	–	2	–	1	2	2
Other^****^	5	3	2	6	4	7	5	5

*Question: “Have you received support outside the hospital for work-related problems?”

**Question: “Have you visited other healthcare professionals for support with work-related problems in the past three months?”

***Intervention group, e.g. occupational physician, employer, social security agency (UWV), general practitioner, cancer care consultant, psychologist. Control group, e.g. occupational physician, physiotherapist, psychologist, financial advisor, vitality coach

****Intervention group, e.g. colleagues, general practitioner, oncological occupational physician, psychologist, physiotherapist, social security agency (UWV).Control group, e.g. manager, employer, labor expert, corporate counselor, psychologist (incl. sports psychologist), oncological nurse, general practitioner

Alongside the rather broad range of RTW rates described in the systematic review conducted by Mehnert [48], a few other studies have also reported on the length of sick leave (i.e. days until RTW). In this respect, too, our study reveals considerable differences. Some patients did not report sick at all, whereas others had still not returned to work twelve months after baseline. However, the systematic review of Mehnert [48] was based on 64 studies and so many different characteristics were included. A mean duration of 151 days of sick leave was reported [48]. Considerable differences in this outcome are also described in the other literature; for example, one study reported a mean of 349 days [49] and another 86 days [50]. These differences are related to a number of factors, such as cancer site, treatment type, physical complaints like level of fatigue, and the level of workload [44]. Because of this, it is complicated to compare the RTW rates and/or days until RTW of patients diagnosed with cancer. In other words, these differences are of importance to the outcome and differ per individual. Although some factors are non-modifiable, it is important to identify those patients ‘at risk’, as also determined by Kiasuwa et al. [51]. All of which further underlines the fact that an intervention must be tailored to the needs of the patient concerned. A ‘one-size-fits-all’ intervention inevitably disregards these different factors. To identify those patients at risk and to tailor the support, we developed a decision diagram with three kind of supports; support A (mild work related problems), B (severe work-related problems) and C (complex work-related problems), respectively. From the results of our study, no patients needed the support of a multidisciplinary team (support C). However, we could not conclude that this support is therefore not needed.

Still, we think that this option must be available, as this was discussed in the development of the intervention with an expert panel [28]. In line with this the clinical relevance of a multi-disciplinary approach [52] and the opinions of the expert panel support C is of value.

The intervention in practice was scored with different key components; recruitment, context, reach, dose delivered, dose received and fidelity. Although the study protocol for the intervention in practice was easy to follow according to the healthcare professionals, there are mainly logistical issues that needs to be revised. Particularly the duration and timing of the intervention resulted in a low fidelity score (protocol adherence) for the oncological occupational physician, while the oncological nurses scored better in the total fidelity score. One of the limitations of the intervention in practice which must be taken into account is that the intervention patients were spread over 16 hospitals which might have caused heterogeneity The inclusion of 16 hospitals resulted in nurses in each hospital not having many work-related support meetings. It is possible that meetings therefore were not completely conducted as intended. The majority of the patients were satisfied and found the intervention useful and the healthcare professionals acknowledge the importance of the awareness of work- related support with the clinical setting.

### Strengths and Limitations

One strength of our intervention study is that we provided ‘tailored’ work-related support, which is an innovative aspect within psychosocial work-related support. At the present time there are more studies about RTW interventions; however, the intervention we developed was also innovative in that it supported patients with work-related problems from an early stage of their diagnosis. Supporting patients with work-related issues in the clinical setting is a subject acknowledged as important by the participating healthcare professionals in this study, although it is still rarely discussed in such an early phase [17, 18].

Another strength of the intervention was the involvement of different healthcare professionals to ensure tailored work-related support. The oncological nurse is the first professional in a position to discuss work with the patient, to notice possible (experienced) work-related problems, and possibly also to involve the second important healthcare professional: the oncological occupational physician (OOP), who possesses specialized knowledge about cancer in relation to work [29]. The OOP is involved in the clinical setting instead of being employed by an occupational health service, which makes them even more independent to patients’ perception.

On the other hand, one major limitation of this study—as already mentioned—was its inability to include a sufficient number of patients. Another was the differences in the treatment of the different GI diagnoses, because these could influence RTW. At baseline, however, the intervention and control groups were equal in respect of their diagnoses.

### Study Design Considerations

This study was performed as a randomized controlled trial (RCT), a research method whereby participants are assigned randomly to a group so as to preclude differences between groups. Nevertheless, in this case two factors might have caused selection bias. First, healthcare professionals may not have informed some eligible patients about the study, thus resulting in the number of participating patients being rather low. If this was the case, one possible explanation is that patients nowadays are often asked to participate in more than one study; however, they should not be overloaded with such requests, especially when they have just been diagnosed with cancer. Petersen et al. [18] point out that occupational support is not yet integrated with clinical care and so, despite interest in this kind of work-related support, healthcare providers could be critical towards patient recruitment. Unfortunately, we have no data concerning all the eligible patients because this was not recorded efficiently at the participating hospitals. Moreover, those who are included in the study are likely to be ones willing to participate because they know the value of research and are interested in the subject.

### Recommendations for Research and Practice

Work-related support is an issue which is becoming increasingly recognized in day-to-day clinical and psycho-oncological practice. However, it is recommended that research continue in order to develop a consistent foundation and better knowledge whereby healthcare professionals can support patients (in an early phase) with work-related issues [18, 53, 54]. Another recommendation for future research, when the intervention is proven effective, is to perform an economic evaluation from a societal perspective including costs of intervention, loss of productivity and costs of sick leave days. Although no statistically significant differences were found in this study, the results are still interesting: a difference of 43 days less sick leave for the intervention group, compared with the ‘care as usual’ control group, plus the positive experiences reported by both patients and healthcare professionals. The value of an economic evaluation is important showing that tailored work-related support is beneficial for the individual patient as well as for society at large will provide more knowledge and generate greater urgency to implement such support in day-to-day practice.

### Generalizability

Because we used a multicenter RCT design and included 16 different hospitals distributed over the Netherlands, we can interpret the results of this study as applicable to the Netherlands as a whole. Individuals who become ill are protected by the social security system in the Netherlands. This Act is called the ‘Improved Gatekeepers Act’, is active for the first two years of sick leave and partly covers the wage loss. Both employee and employer need are obliged to actively insert effort for the return to work process. The occupational physician also takes part in this process as well. Generalizability to other countries is possible if their clinical healthcare provision is comparable with the situation described in this study. It should be noted, however, that we used OOPs in our study: physicians specialized in occupational support for oncology-related issues [29]—a specific type of healthcare professional that may not available everywhere, or whose role and remit could be different in other countries.

### Conclusion

Our analysis shows a possible positive effect of the intervention on enhancement of RTW. The results add novel findings about early, tailored work-related support in the clinical setting.
